# Characterization and Genomic Study of the Novel Bacteriophage HY01 Infecting Both *Escherichia coli* O157:H7 and *Shigella flexneri*: Potential as a Biocontrol Agent in Food

**DOI:** 10.1371/journal.pone.0168985

**Published:** 2016-12-30

**Authors:** Heyn Lee, Hye-Jin Ku, Dong-Hoon Lee, You-Tae Kim, Hakdong Shin, Sangryeol Ryu, Ju-Hoon Lee

**Affiliations:** 1 Department of Food Science and Biotechnology and Institute of Life Science and Resources, Kyung Hee University, Yongin, Republic of Korea; 2 Department of Food and Animal Biotechnology, Department of Agricultural Biotechnology, Research Institute of Agriculture and Life Sciences, Seoul National University, Seoul, Republic of Korea; 3 Faculty of Food Science and Biotechnology, College of Life Science, Sejong University, Seoul, Republic of Korea; Centro Nacional de Biotecnologia, SPAIN

## Abstract

**Background:**

*Escherichia coli* O157:H7 and *Shigella flexneri* are well-known food-borne pathogens causing severe food poisoning at low infectious doses. Bacteriophages have been approved for food applications by the US Food and Drug Administration (FDA) and have been suggested as natural food preservatives to control specific food-borne pathogens. To develop a novel natural food preservative against *E*. *coli* O157:H7 and *S*. *flexneri*, a new bacteriophage needs to be isolated and characterized.

**Methodology/Principal Findings:**

Bacteriophage HY01 infecting both *E*. *coli* O157:H7 and *S*. *flexneri* was isolated from a swine fecal sample. HY01 belongs to the family *Myoviridae* and is stable under various temperature and pH conditions. One-step growth curve analysis showed relatively short eclipse and latent periods as well as large burst size. The 167-kb genome sequence of HY01 was sequenced, and a comparative genome analysis with T4 for non-O157:H7 *E*. *coli* suggests that the receptor recognition protein of HY01 plays an important role in determination of host recognition and specificity. In addition, food applications using edible cabbage were conducted with two *E*. *coli* O157:H7 strains (ATCC 43890 and ATCC 43895), showing that treatment with HY01 inhibits these clinical and food isolates with >2 log reductions in bacterial load during the first 2 h of incubation.

**Conclusions/Significance:**

HY01 can inhibit both *E*. *coli* O157:H7 and *S*. *flexneri* with large burst size and stability under stress conditions. The ability of HY01 to infect both *E*. *coli* O157:H7 and *S*. *flexneri* may be derived from the presence of two different host specificity-associated tail genes in the genome. Food applications revealed the specific ability of HY01 to inhibit both pathogens in food, suggesting its potential as a novel biocontrol agent or novel natural food preservative against *E*. *coli* O157:H7 and potentially *S*. *flexneri*.

## Introduction

*Escherichia coli* O157:H7 is one of the major food-borne pathogens causing enteric illnesses including abdominal cramps, diarrhea, hemolytic colitis, hemolytic uremic syndrome (HUS), and even kidney failure in humans, with an estimated minimum infectious dose of 10^1^ to 10^2^ CFU [1]. *Shigella flexneri* is another wide-spread food-borne pathogen causing human diarrhea termed “shigellosis.” These bacteria are responsible for food poisoning outbreaks worldwide through various foods, such as ground meat, raw milk, apple cider, and fresh vegetables. In 2011, about 2,140 hospitalizations due to food-borne *E*. *coli* O157:H7 and approximately 500,000 cases of shigellosis were reported in the United States [2]. Although antibiotics were the most effective treatment agents to control bacterial pathogens, abuse of antibiotics has caused the emergence of antibiotic-resistant strains. As such, the US FDA has disallowed the use of antibiotics for bacterial control in foods, and the use of chemical food preservatives is not preferred. Therefore, natural biocontrol agents for food preservation have been suggested for use in foods for the prevention of food-borne pathogen contamination, including bacteriocins, organic acids, ethanol, chitosan oligosaccharides, lactoferrin, and even bacteriophages.

Bacteriophages are viruses that infect specific bacteria via both lysogenic and lytic life cycles [3]. During the lytic cycle, bacteriophages lyse and disrupt bacterial cells, indicating bactericidal activity. Although bacteriophages have been used for therapeutic purposes in Eastern Europe, the broad application of various antibiotics in Western countries has replaced the use of bacteriophages [4]. However, the emergence of antibiotic-resistant pathogens and problematic food-borne pathogens has recently re-attracted public interest in the use of bacteriophages for pathogen control. In particular, bacteriophages can infect specific bacteria without any effect on normal flora and without any side effects for human applications [1]. Furthermore, the US FDA approved the use of bacteriophages as a novel food preservative in 2006 (ListShield^™^). Since then, a number of phage products have been developed in the US for pathogen control, including that of *E*. *coli* O157:H7 (EcoShield^™^ and Finalyse^™^), *Salmonella* (SALMONELEX^™^, BIOTECTOR S1, and BIOTECTOR S4), and *Listeria* (LISTEX^™^ P100).

Recently, bacteriophages have been tested as novel natural food preservatives for various fresh vegetables and meat products contaminated with *E*. *coli* O157:H7 or *S*. *flexneri*. Use of the phage ECP-100 to treat ground beef contaminated with *E*. *coli* O157:H7 showed up to a 1.28 log reduction in bacterial load after 24 h and up to 1.96 log reduction after a 168 h incubation [5]. In addition, use of phage FAHEc1 to treat raw beef containing *E*. *coli* O157:H7 revealed a 4.5 log reduction in bacterial load, and this inhibition activity was sustained for 25 h [6]. *S*. *flexneri* phage SF-A2 used in Ready-To-Eat sliced chicken showed a 2 log reduction over a 72 h incubation [7]. To improve bacterial host growth inhibition by phage, an *E*. *coli* O157:H7 phage cocktail containing three distinct phages (e11/2, e4/1c, and pp01) was developed. Use of the phage cocktail to treat a steak meat sample contaminated with *E*. *coli* O157:H7 showed an about 5 log reduction in bacterial load after up to an 8 h incubation, suggesting that the phage cocktail may be more effective than single phages alone for pathogen growth inhibition in foods [8]. Taken together, these data suggest that *E*. *coli* O157:H7 and *S*. *flexneri* phages work for the reduction of bacterial host cells in foods.

Next-generation sequencing (NGS) technologies have allowed for comparative phage genomic studies. In general, phage genome analyses have revealed that phage genes belong to several functional groups including DNA replication/manipulation, transcription, host lysis, structure and packaging, host recognition, and additional functions [9]. In particular, phage genome information has provided insight into the host-phage interaction and host specificity via analysis of tail fiber and/or tail spike proteins. As an example, comparative genome analysis of the phages T4 infecting *E*. *coli* and AR1 infecting *E*. *coli* O157:H7 revealed that their genome sequence shares >98% DNA sequence identity, but the DNA sequences encoding tail fiber proteins in the two phage genomes are quite different, supporting their different host specificity [10, 11]. Therefore, phage genome information will be useful to expand our understanding of phage characteristics and their recognition of and interaction with specific host strains at the genomic level.

In this study, a newly isolated phage HY01 was characterized using the host range test, challenge assay, one-step growth curve analysis, and stability tests under various stress conditions to verify if this phage has potential as a novel biocontrol agent of *E*. *coli* O157:H7 and *S*. *flexneri* pathogens in foods. In addition, the HY01 genome was sequenced and analyzed to further understand the characteristics of this phage at the molecular level.

## Materials and Methods

### Bacterial strains and growth conditions

Bacterial strains and growth media used in this study are listed in Table 1. All bacteria were routinely cultivated with shaking at 37°C. *E*. *coli* O157:H7 ATCC 43890 and *S*. *flexneri* 2a strain 2457T were selected as indicator strains for phage isolation.

**Table 1:** Host range of phage HY01.

Bacterial strains	Plaque formation^a^	Source or reference^b^	Medium^c^
*E*. *coli* O157:H7			
ATCC 35150	+	ATCC	LB
ATCC 43888	+	ATCC	LB
ATCC 43890	+	ATCC	LB
ATCC 43894	+	ATCC	LB
ATCC 43895	+	ATCC	LB
*Shigella flexneri*			
2a strain 2457T	+	IVI	LB
NCTC 8519	+	NCTC	LB
ATCC 29903	+	ATCC	LB
ATCC 12022	+	ATCC	LB
*E*. *coli*			
K-12 MG 1655	-	[29]	LB
DH5α	-	Invitrogen	LB
*Salmonella* Typhimurium			
SL1344	-	NCTC	LB
LT2	-	[30]	LB
*S*. Enteritidis ATCC 13076	-	ATCC	LB
*Cronobacter sakazakii* ATCC 29544	-	ATCC	TSB
*Yersinia enterocolitica* ATCC 29544	-	ATCC	TSB
*Pseudomonas aeruginosa* KACC 10186	-	KACC	TSB
*Bacillus cereus* ATCC 13061	-	ATCC	BHI
*B*. *subtilis* ATCC 23857	-	ATCC	BHI
*Listeria monocytogenes* Scott A	-	[31]	BHI
*Enterococcus faecalis* ATCC 29212	-	ATCC	MRS
*Staphylococcus epidermidis* KACC 13234	-	KACC	TSB
*S*. *aureus* ATCC 29213	-	ATCC	TSB

^a^, +, susceptible to HY01; -, not susceptible to HY01

^b^, ATCC, American Type Culture Collection; NCTC, National Collection of Type Cultures; KACC, Korean Agricultural Culture Collection; IVI, International Vaccine Institute.

^c^, LB, Lysogeny Broth; BHI, Brain Heart Infusion; TSB, Tryptic Soy Broth; MRS, de Man-Rogosa-Sharpe.

### Bacteriophage isolation and propagation

A swine fecal sample collected from a farm at Seoul National University in Suwon, South Korea, was used for bacteriophage isolation. *E*. *coli* O157:H7 ATCC 43890 was used as the indicator strain for phage isolation, purification, and propagation. The phage isolation was performed using previously described procedures [12]. For phage purification, a pure single plaque was picked after five repetitive single-plaque formation processes [9]. After serial phage propagations, the phages were concentrated using precipitation with polyethylene glycol (PEG) 6000 (Daejung, Siheung, Korea) and purified using CsCl ultracentrifugation [9]. The purified phages were stored at 4°C.

### Electron microscopy

Purified HY01 phage morphology was observed and determined using energy-filtering transmission electron microscopy (EF-TEM; JEM1010, JEOL, Tokyo, Japan). The TEM staining and analysis procedures were performed as previously described [12]. Morphological classification was performed according to the guidelines of the International Committee on Taxonomy of Viruses (ICTV).

### Host range test

The individual test strain was added to 6 ml of molten 0.4% soft agar and then overlaid on 1.8% agar plates. Ten-fold serially diluted HY01 was plated on the agar plates containing the host strain. After incubation at 37°C for 12 h, the number of plaques were counted to determine HY01 phage titer and plaque forming unit (PFU) calculation. In addition, efficiency of plating (EOP) was determined by comparison of the phage titer of the test strain with that of *E*. *coli* O157:H7 ATCC 43890 as a reference strain.

### One-step growth curve

Thirty-milliliter cultures of *E*. *coli* O157:H7 ATCC 43890 and *S*. *flexneri* 2a strain 2457T were incubated until an optical density (OD) of 1.0 was reached at 600 nm (4 X 10^7^ CFU/ml for *E*. *coli* O157:H7 and 10^9^ CFU/ml for *S*. *flexneri*), at which time the bacterial cells in the culture were harvested. Phage HY01 was added at a multiplicity of infection (MOI) of 0.001 and was allowed to adsorb to the host strain *E*. *coli* O157:H7 ATCC 43890 or *S*. *flexneri* 2a strain 2457T for 20 min at room temperature. After phage adsorption, the mixture was centrifuged at 6000 × *g* for 10 min to remove non-absorbed phage in the supernatant. The pellet was resuspended with 50 ml of fresh LB broth and incubated with shaking at 37°C. Two sets of samples were collected every 5 min, and 1% chloroform (final concentration) was added to the second of the two sets to release intracellular phage. Then, the two samples were serially diluted 10-fold and plated for phage titration. Based on the comparison of PFU/ml between the chloroform-treated and non-treated sets of the samples, the eclipse/latent periods and burst size were determined.

### Stability test under various stress conditions

To determine phage stability under various pH conditions, the sodium chloride-magnesium sulfate (SM) buffer (100 mM NaCl, 10 mM MgSO_4_ 7H_2_O, and 50 mM Tris HCl, pH 7.5; Sigma, St. Louis, MO) pH was adjusted with 4 N HCl or 2 N NaOH over a pH range of 1 to 12, and the phage HY01 was added at a final concentration of 10^9^ PFU/ml. After incubation at 37°C for 12 h, the phage suspensions were neutralized, and phage titers were determined using plaque assays with *E*. *coli* O157:H7 ATCC 43890 as a reference strain. To determine phage stability under various temperature conditions, the phage HY01 (final concentration, 10^9^ PFU/ml) was added to SM buffer and incubated at -20, 20, 30, 37, 40, 50, 60, 65, and 70°C for 12 h. After incubation, phage titers were determined with plaque assays using the same reference strain [13].

### Bacterial challenge test

*E*. *coli* O157:H7 ATCC 43890 and *S*. *flexneri* 2a strain 2457T were incubated with shaking at 37°C for 12 h, and a subsequent 1% inoculation was transferred to 100 ml fresh LB broth. After incubation at 37°C with shaking until the OD_600_ reached 1.0, the culture was divided into two 50-ml cultures. Phage HY01 at a MOI of 10 was added to one of the cultures, and the two samples were incubated with shaking at 37°C. Samples were collected every hour, serially diluted 10-fold, and plated on LB agar plates. After incubation at 37°C, the colony forming units (CFU)/ml of each sample was determined using a viable cell count method.

### Genome sequencing and bioinformatics analysis

Genomic DNA extraction of HY01 was carried out as described previously [14] and sequenced by next generation sequencing (NGS) technology using a Genome Sequencer FLX (GS-FLX) Titanium (Roche, Mannheim, Germany). The qualified sequence reads were assembled using Newbler v2.3 (Roche). The open reading frames (ORFs) were predicted using Glimmer3 [15], FgenesV (SoftBerry, Inc., Mount Kisco, NY), and GeneMarkS [16], and the ribosomal binding sites (RBSs) were predicted using RBSfinder for confirmation of ORF predictions (J. Craig Venter Institute, San Diego, CA). Functions of the predicted ORFs were annotated using BLASTP [17] and InterProScan programs with protein domain databases [18]. Sequence alignments were conducted using MEGA6 [19] and BLAST2SEQ programs [20]. The genome sequence was handled and edited using Artemis16 [21], and comparative genome analysis was performed using ACT12 [22] and JDotter [23].

### Proteomic analysis of phage structural proteins

To analyze the phage protein profile, phage HY01 (10^10^ PFU/ml) was boiled for 5 min, and the denatured phage proteins were separated using SDS-polyacrylamide gel electrophoresis (SDS-PAGE) with Mini-PROTEAN TGX Precast gels (Bio-Rad, Hercules, CA). Among the separated protein bands, five major protein bands were excised and recovered from the gel. The molecular weights of those proteins were determined using MALDI-TOF/MS. Mass spectrometry was conducted using the AB SCIEX TOF/TOFTM 5800 system (Framingham, MA) in positive reflector mode by the Korea Basic Science Institute (KBSI; Seoul, Korea). In MS mode, scan parameters of mass range and total number of laser shots were 800–4,000 Da and 400, respectively. The selected precursors were fragmented at 1-kV collision energy with air, and the metastable suppressor mode was selected. The ProteinPilot 4.0 program using the Paragon algorithm (AB SCIEX) was used for protein identification.

### Food application

To verify the ability of phage HY01 to inhibit host bacteria in food environments, a clinical *E*. *coli* isolate (*E*. *coli* O157:H7 ATCC 43890; 10^3^ CFU in 0.1 ml) or a food *E*. *coli* isolate (ATCC 43895; 10^3^ CFU in 0.1 ml) was applied and spread on the surface of five square pieces (5 g) of fresh edible cabbage. After application of the test strains, phage HY01 was sprayed on the pieces of cabbage at MOIs of 10^5^ or 10^6^. Then, the pieces were transferred into five different culture tubes, the cabbage samples were subsequently incubated at 37°C, and the test samples were collected at 0, 1, 2, 4, and 6 h incubation time points. After collection of the samples, the bacterial cells were separated and collected using stomaching with 45 ml of sterilized 0.1% peptone water for 30 sec. After stomaching, the cabbage debris was removed using centrifugation at 2,000 rpm for 1 min, and the supernatants were transferred to new centrifuge tubes. The bacterial cells were collected at 6,000 rpm for 10 min, and the bacterial cell pellets were resuspended and serially diluted 10-fold with fresh LB broth. Colonies on triplicate Eosin Methylene Blue (EMB) agar plates were counted, and this viable cell count was used to determine HY01 inhibition activity.

## Results

### Host range and morphological observation of phage HY01

**Fig 1:**
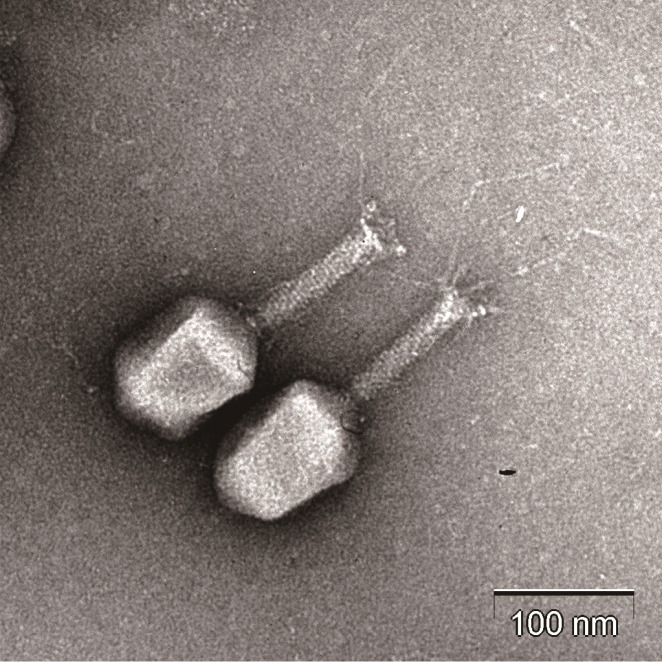
TEM image of phage HY01. Scale bar, 100 nm.

To control the food-borne pathogens *E*. *coli* O157:H7 and *S*. *flexneri*, a novel HY01 phage was isolated from a swine fecal sample using *E*. *coli* O157:H7 ATCC 43890 as an indicator strain. Interestingly, this phage specifically infects various *E*. *coli* O157:H7 strains and *S*. *flexneri* strains, but does not infect other types of *E*. *coli* or other bacteria including *Bacillus*, *Listeria*, *Salmonella*, *Pseudomonas*, *Enterococcus*, *Staphylococcus*, *Cronobacter*, or *Yersinia*, suggesting that this phage has high host specificity to *E*. *coli* O157:H7 and *S*. *flexneri* (Table 1). TEM analysis revealed that phage HY01 has an icosahedral head, a contractile and nonflexible tail, and tail fibers, suggesting that it belongs to the family *Myoviridae*, which is characterized by an approximately 100-nm icosahedral head and a 100-nm tubular tail with tail fibers (Fig 1).

### One-step growth curve

**Fig 2:**
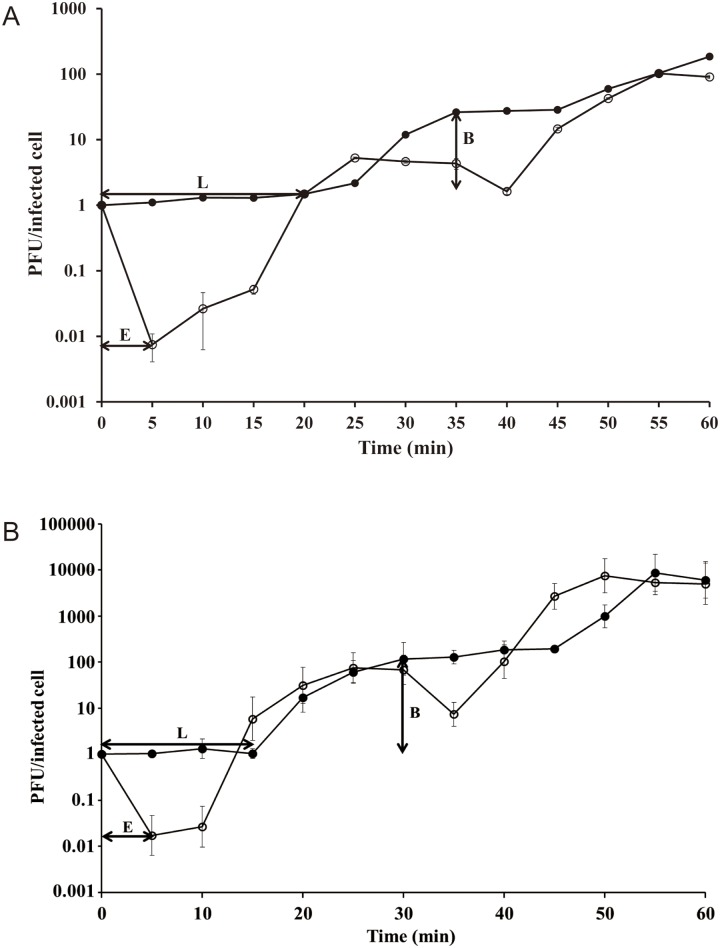
One-step growth curve analysis of phage HY01. (A) *E*. *coli* O157:H7 ATCC 43890 and (B) *S*. *flexneri* 2a strain 2457T as host strains. Open circles, chloroform-treated samples; closed circles, non-chloroform-treated samples. E, eclipse period; L, latent period; B, burst size. The error bars indicate standard deviations.

To determine the eclipse period, latent period, and burst size of bacteriophage HY01, one-step growth curve analysis was conducted with *E*. *coli* O157:H7 ATCC 43890 and *S*. *flexneri* 2a strain 2457T as the indicator strains (Fig 2). This analysis showed 5 min and 25 min for the eclipse period and latent period with *E*. *coli* O157:H7, respectively, and 5 min and 15 min with *S*. *flexneri*. In the test strains of *E*. *coli* O157:H7 and *S*. *flexneri*, the burst sizes were about 25 and 100 PFU per infected cell, respectively.

### Phage stability under stress conditions

**Fig 3:**
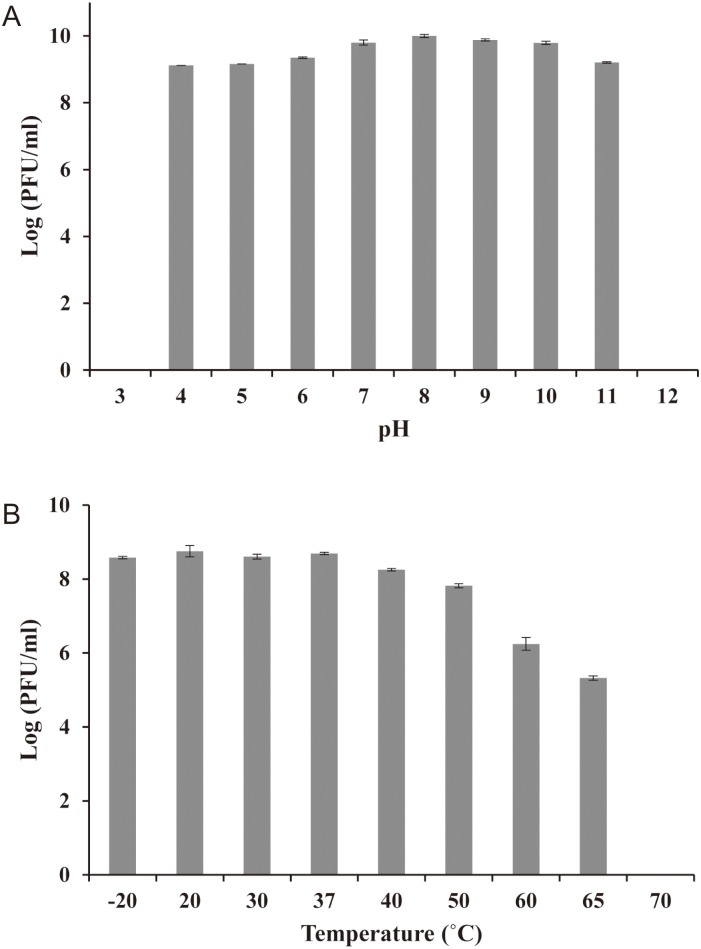
Stability of phage HY01 under various stress conditions. (A) pH stability and (B) temperature stability. *E*. *coli* O157:H7 ATCC 43890 was used as the host strain.

For broad applications in food, the phage should be stable under various stress conditions including temperature and pH. To determine the stability of phage HY01, its stability was tested under a broad range of temperatures (-20 to 70°C) and pH values (1 to 12). Interestingly, phage HY01 showed stability under various temperatures (-20 to 65°C) and pH levels (4 to 11), suggesting that this phage is suitable for food applications (Fig 3). However, the viability of phage HY01 was completely abolished under strong acidic and alkaline conditions (<3 or >12) as well as at high temperature (>70°C).

### Bacterial challenge assay

**Fig 4:**
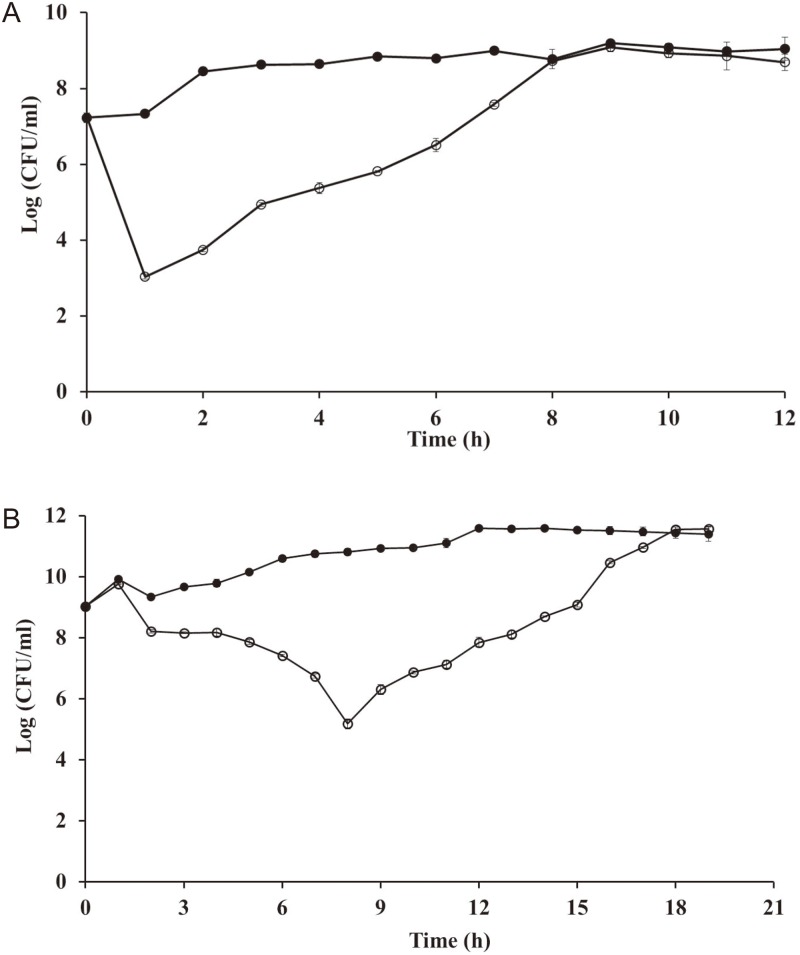
Bacterial challenge assay of phage HY01. (A) *E*. *coli* O157:H7 ATCC 43890 and (B) *S*. *flexneri* 2a strain 2457T as host strains. Open circles, HY01-infected samples; closed circles, non-infected samples.

Bacterial host lysis and bacteriophage insensitive mutants (BIM) of HY01-sensitive *E*. *coli* O157:H7 and *S*. *flexneri* were determined by growth curve analysis and viable-cell counts after HY01 infection (Fig 4). These two indicator strains were cultivated up to early stationary phase (OD_600_ = 1.0), and then phage HY01 was added to the cultures. The assay with *E*. *coli* O157:H7 ATCC 43890 revealed a 4 log reduction in bacterial load at 1 h, and the growth recovery of the strain was completed within 8 h. However, the assay with *S*. *flexneri* 2a strain 2457T showed a 4 log reduction at 8 h, and the growth recovery of the strain was completed within 18 h. Interestingly, while the *E*. *coli* O157:H7 indicator strain was highly sensitive to phage infection and the BIM emerged shortly thereafter, the growth inhibition activity of *S*. *flexneri* indicator strain with HY01 was well-sustained, and the BIM occurred slowly, suggesting that HY01 will be useful for phage applications against *S*. *flexneri*.

### Genomic characterization

**Fig 5:**
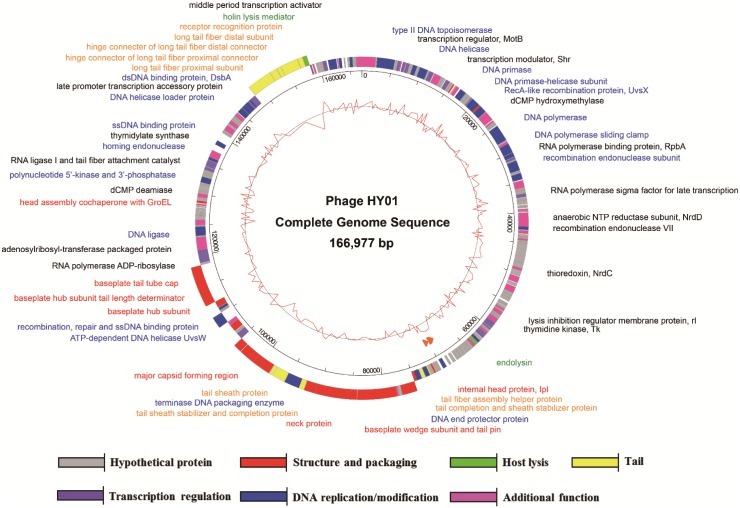
Genome map of phage HY01.

To understand the phage infection and host-phage interaction at the molecular level and to confirm the absence of toxin genes and virulence factors for food applications, the complete genome sequence of phage HY01 was determined. Analysis revealed that the HY01 genome consists of 166,977 nt with a GC content of 35.5%, containing 258 predicted open reading frames (ORFs) and 9 tRNA genes (Table 2). Among the predicted ORFs, 144 (55.8%) were predicted to have specific functions. These functional ORFs were categorized into six functional groups: DNA replication/modification (Type II DNA topoisomerase, DNA helicase, DNA primase, and DNA polymerase), transcription regulation (transcription regulator MotB, RNA polymerase binding protein, RNA polymerase sigma factor, and RNA polymerase ADP-ribosylase), host lysis (endolysin and holin lysis mediator), structure and packaging (major capsid protein, baseplate wedge, neck protein, and internal head protein), tail (short tail fiber, tail sheath protein, long tail fiber, and receptor recognition protein), and additional functions (thymidylate synthase, dihydrofolate reductase, and dCMP deaminase) (Fig 5).

**Table 2:** General genome features of phage HY01 and other closely related phages.

Genome characteristics	Phage				
	HY01	T4	AR1	pSs-1	Shfl2
Genome size (bp)	166,977	168,903	167,435	164,999	165,919
G+C%	35.5	34.5	35.3	35.54	35.57
Predicted ORFs	257	289	281	266	265
tRNAs	9	8	10	10	10
GenBank accession no.	KF925357	AF158101	AP011113	KM501444	HM035025

### Comparative genome analysis

**Fig 6:**
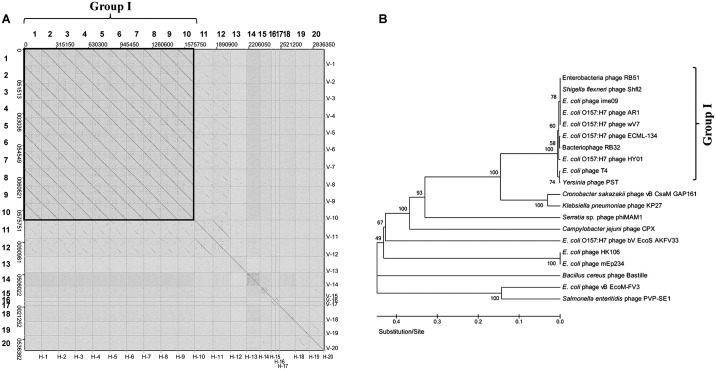
Comparative genome analysis of phage HY01 and 19 closely related or similar phages. (A) Dot plot alignment of 20 phage genome sequences. 1. Phage HY01, 2. *E*. *coli* phage RB51 (FJ839693), 3. *E*. *coli* O157:H7 phage ECML-134 (JX128259), 4. *E*. *coli* O157:H7 phage AR1 (AP011113), 5. *Yersinia* phage PST (KF208315), 6. *S*. *flexneri* phage Shfl2 (HM035025), 7. *E*. *coli* phage T4 (AF158101), 8. *E*. *coli* phage ime09 (JN202312), 9. *E*. *coli* phage RB32 (DQ904452), 10. *E*. *coli* O157:H7 phage wV7 (HM997020), 11. *Cronobacter sakazakii* phage vB_CsaM_GAP161 (JN882287), 12. *Klebsiella pneumoniae* phage KP27 (HQ918180), 13. *Serratia* sp. phage phiMAM1 (JX878496), 14. *Campylobacter jejuni* phage CPX (JN132397), 15. *E*. *coli* O157:H7 phage bV_EcoS_AKFV33 (HQ665011), 16. *E*. *coli* phage HK106 (JQ086369), 17. *E*. *coli* phage mEp234 (JQ182732), 18. *Bacillus cereus* phage Bastille (JF966203), 19. *E*. *coli* phage vB_EcoM-FV3 (JQ031132), 20. *Salmonella* Enteritidis phage PVP-SE1 (GU070616). (B) Phylogenetic tree analysis of terminase large subunits from phage HY01 and 19 phages using the neighbor-joining method. Bootstrap values are indicated at the branches of the tree. Group I phages are indicated with a bracket.

To determine phage HY01-specific genome characteristics, its genome was compared with closely related phage genomes using dot plot alignments, phylogenetic tree analysis, and whole genome multiple sequence alignments. Dot plot alignment of HY01 with 19 closely related or similar phages showed that phage HY01 is highly homologous to nine other phages at the DNA sequence level, which were designated as Group I (Fig 6A). To verify this result, subsequent phylogenetic tree analysis of all 20 selected phages using the protein sequences of the terminase large subunit was conducted. Interestingly, Group I was also detected in the phylogenetic tree, supporting the dot plot result (Fig 6B). The hosts in Group I are *E*. *coli*, *E*. *coli* O157:H7, *S*. *flexneri*, and *Yersinia*, suggesting that their phages are closely related and may have evolved from a common ancestor.

**Fig 7:**
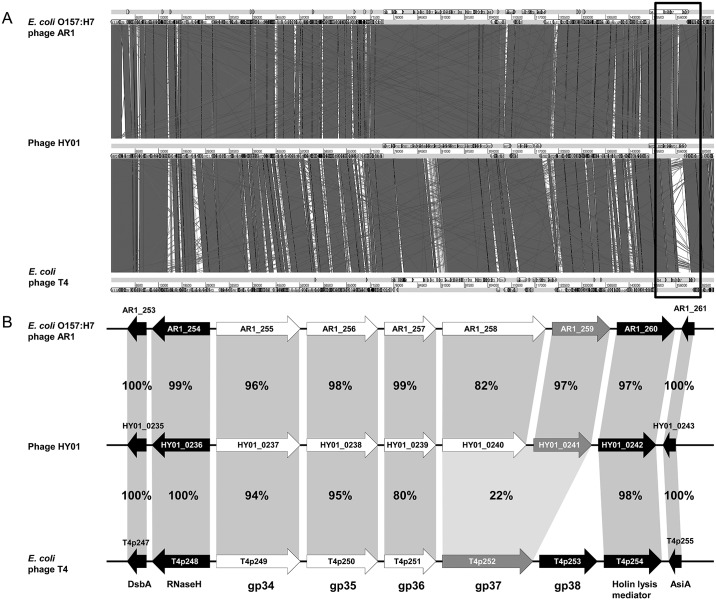
Comparative genomic analysis of phage HY01 with two closely related *E*. *coli* phage genomes (AR1 and T4). (A) Whole genome alignment of three phage genomes using ACT13. The yellow open box indicates a small gene cluster with low homology between the HY01 and T4 genomes. (B) Organization of a small gene cluster from the yellow open box. The white arrows indicate long tail fiber-associated genes, the blue arrows indicate receptor recognition genes, and the black arrows indicate genes with other functions.

**Fig 8:**
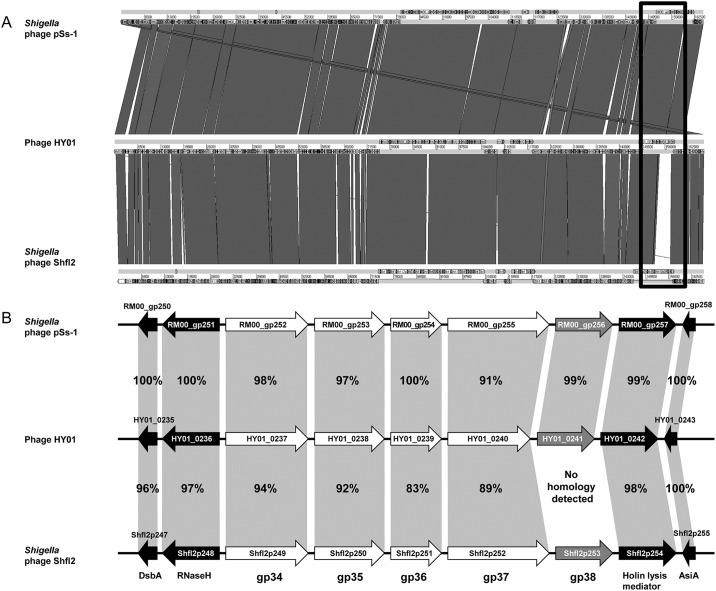
Comparative genomic analysis of phage HY01 with two closely related *Shigella* phage genomes (pSs-1 and Shfl2). (A) Whole genome alignment of three phage genomes using ACT13. The yellow open box indicates a small gene cluster with low homology between the HY01 and Shfl2 genomes. (B) Organization of a small gene cluster from the yellow open box. The white arrows indicate long tail fiber-associated genes, the blue arrows indicate receptor recognition genes, and the black arrows indicate genes with other functions.

To further understand HY01-specific genome characteristics, the entire HY01 phage genome was compared with two closely related *E*. *coli* phage genomes (AR1 and T4) and two *Shigella* phage genomes (pSs-1 and Shfl2). Genome comparison of HY01 and the two other *E*. *coli* phage genomes revealed that *E*. *coli* O157:H7 phage AR1 and *E*. *coli* phage T4 have 95.2% and 94.8% sequence identity, respectively, at the DNA level (Fig 7A). However, genome comparison between HY01 and T4 showed a small non-matched gene cluster containing two genes encoding a long tail fiber distal subunit (HY01_0240/ AR1_258) and receptor recognition protein (HY01_0241/AR1_259). The functions of these two genes may be involved in specific host recognition and specificity for phage infection. Furthermore, genome comparison of HY01 and two other *Shigella* phage genomes showed that *Shigella* phage pSs-1 and *S*. *flexneri* phage Shfl2 have 96.6% and 95.8% sequence identity, respectively, at the DNA level (Fig 8A). Similar to the genome comparison of HY01 and *E*. *coli* phage T4, a small gene cluster was detected that differed between the HY01 and Shfl2 genomes. In this small gene cluster, the long tail fiber distal subunits (HY01_0240/Shfl2p252) had high sequence identity (89%), but the receptor recognition proteins had no sequence identity, which is different from the small gene cluster between the HY01 and T4 genomes (Fig 8B). Nevertheless, the HY01 and Shfl2 phages infect the same species, *S*. *flexneri*.

### Proteomic analysis of phage structural proteins

**Fig 9:**
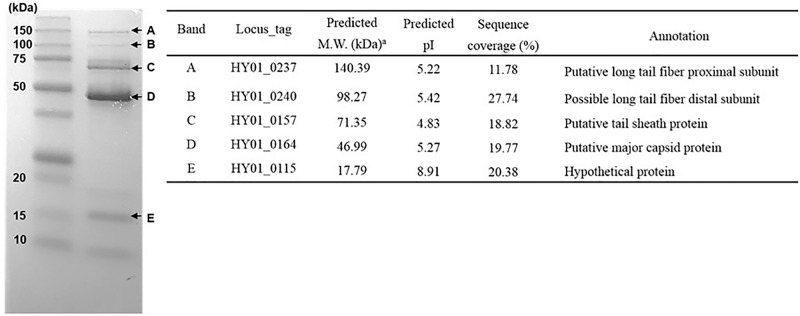
Proteomic analysis of phage structural proteins in HY01 using SDS-PAGE and MALDI-TOF/MS.

A proteomic analysis using SDS-PAGE and MALDI-TOF/MS was performed to determine the phage major protein of HY01. The denatured phage proteins were separated by SDS-PAGE, and five major bands were detected. The partial peptide sequences of the five major bands were analyzed and compared with the genome sequence of phage HY01 for the identification of phage proteins. Four bands were identified as phage structural proteins (a major capsid protein and three phage tail proteins), and one band was identified as a hypothetical protein (Fig 9). In addition, the sizes of the five detected proteins based on SDS-PAGE and their molecular weights determined by MALDI-TOF/MS were matched to their predicted molecular weights from the complete genome sequence analysis data (Fig 5), indicating that most of the detected proteins are associated with phage structure.

### Food application

**Fig 10:**
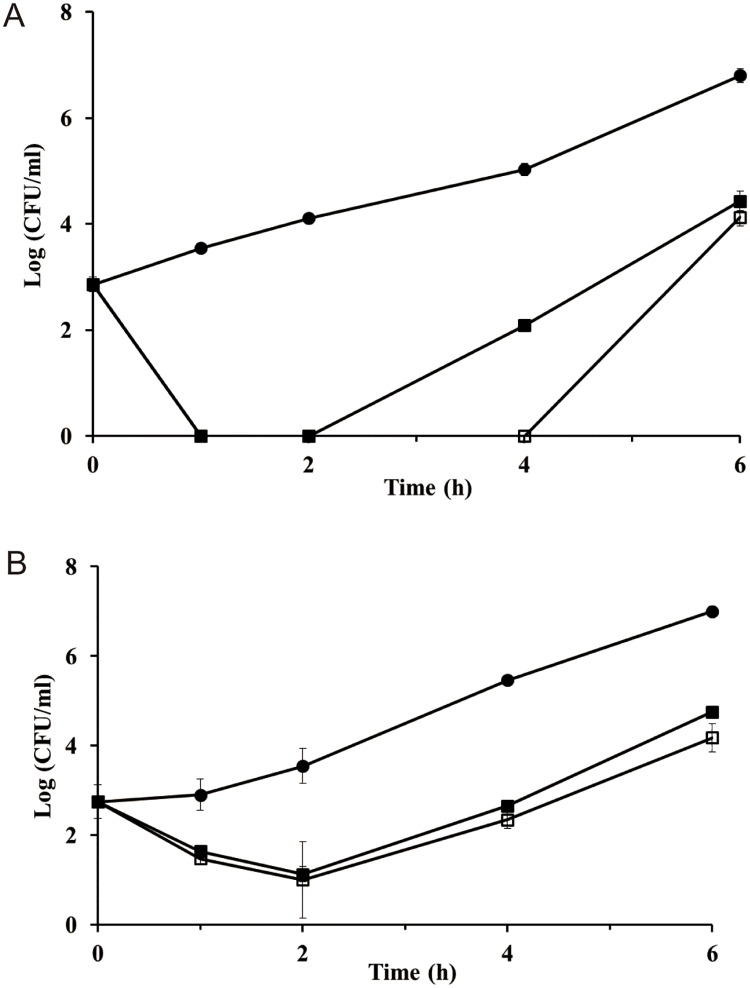
Food application of phage HY01. (A) *E*. *coli* O157:H7 ATCC 43890 (clinical isolate) and (B) *E*. *coli* O157:H7 ATCC 43895 (food isolate) as host strains. Closed circles, control sample without HY01; closed squares, MOI = 10^5^; open squares, MOI = 10^6^.

To determine the potential of HY01 as a novel natural food preservative or biocontrol agent in foods, phage HY01 was added to fresh edible cabbage containing 10^3^ CFU of clinical or food *E*. *coli* O157:H7 isolates, and the number of viable cells in the cabbage were monitored. The phage HY01 at MOIs of 10^5^ and 10^6^ inhibited the clinical *E*. *coli* O157:H7 ATCC 43890 strain, which was not detected after 1 h incubation. Furthermore, with of MOIs of 10^5^ and 10^6^, complete inhibition of the clinical strain was sustained for up to 2 h and 4 h, respectively (Fig 10A). In addition, phage HY01 inhibited the food isolate strain *E*. *coli* O157:H7 ATCC 43895 for up to 2 h at MOIs of 10^5^ and 10^6^. However, HY01 did not completely inhibit the food isolate strain, and growth recovery of the food isolate strain was seen after 2 h incubation, suggesting that the phage HY01 efficiently inhibited the clinical strain longer than the food isolate strain (Fig 10B).

## Discussion

Bacteriophages are bacterial viruses that infect specific hosts for lysis; therefore, phages have been suggested as novel biocontrol agents with host-specific lysis activity. In this study, the novel phage HY01 was isolated and selected because it has host specificity to the well-known food-borne pathogens *E*. *coli* O157:H7 and *S*. *flexneri*. In this study, phage HY01 was biologically characterized, and its genome was completely analyzed for its potential food applications. Our results showed host lysis activity against these food-borne pathogens, high stability under various temperature and pH conditions, and no toxin gene/virulence factors in the HY01 genome, suggesting that HY01 is a potential candidate as a natural food preservative and bacterial growth inhibitor in foods.

While most phages inhibit the growth of a single specific host strain [24], some broad-host range phages that infect different genera or species strains have been reported. Jensen et al. isolated several phages that simultaneously infect two different genera among *Sphaerotilus natans*, *Pseudomonas aeruginosa*, or *E*. *coli* [25]. Phage SFP10 was shown to infect both *Salmonella* Typhimurium and *E*. *coli* O157:H7 at the same time [9]. In addition, phage ΦOT8 infects both *Serratia* sp. and *Pantoea agglomerans* [10]. Furthermore, phage PY100 infects several different species strains of *Yersinia* including *Y*. *enterocolitica*, *Y*. *pseudotuberculosis*, *Y*. *pestis*, *Y*. *intermedia*, *Y*. *kristensenii*, *Y*. *frederiksenii*, and *Y*. *mollaretii* [10]. To understand this broad-host range infection mechanism, the genome of phage SFP10 was completely sequenced and analyzed [9]. The host specificity decision gene cluster containing three genes encoding a tail fiber protein and two tail spike proteins was detected in the phage SFP10. Subsequent comparative genomic analysis and BLASTP analysis revealed that the tail fiber protein is highly specific to *E*. *coli* O157:H7, but the two tail spike proteins are specific to *Salmonella* Typhimurium, suggesting that broad-host range infection is due to the presence of two different host specificity-associated genes in a phage genome [9]. Phage HY01 can simultaneously lyse two different host strains, *E*. *coli* O157:H7 and *S*. *flexneri*, indicating that it is a broad-host range phage with the ability to infect two different genera. However, all five host specificity-associated genes in the host specificity decision gene cluster of HY01 (HY01_0237 to 0241) share high protein sequence identity (>92%) with host specificity-associated genes in both *E*. *coli* O157:H7 phages (wV7 and AR1) and *Shigella* phages (pSs-1 and Shfl2), suggesting that the host specificity-associated proteins encoded by these genes recognize and infect both *E*. *coli* O157:H7 and *Shigella* hosts (Table 3). Therefore, host recognition of the phage SFP10 may differ from that of phage HY01, because two host specificity-associated gene variants are present in phage SFP10 for the simultaneous recognition of two different hosts (*E*. *coli* O157:H7 and *Salmonella* Typhimurium), but all host specificity-associated genes in phage HY01 have dual host recognition for the two different strains (*E*. *coli* O157:H7 and *Shigella*). The broad-host range infection ability of certain phages may be beneficial for the simultaneous biocontrol of different pathogens. If various host specificity-associated genes could be cloned into a phage via genetic engineering, a novel broad-host range phage could be developed for the simultaneous biocontrol of various pathogens.

**Table 3:** Comparative protein sequence analysis of host specificity-associated proteins in a phage HY01 gene cluster.

Locus tag	Predicted function	Location (nt)	BLASTP best matches	Identity^a^	Accession no.
HY01_0237	Putative long tail fiber proximal subunit	148539..152411	Long tail fiber proximal subunit pSs1_00252 [*Shigella* phage pSs-1]	1258/1290 (98%)	KM501444
			Phage long tail fiber proximal subunit F412_gp030 [*E*. *coli* O157:H7 phage wV7]	1243/1290 (96%)	HM997020
HY01_0238	Putative hinge connector of long tail fiber proximal connector	152420..153535	Hinge connector of long tail fiber, proximal connector AR1_256 [*E*. *coli* O157:H7 phage AR1]	363/371 (98%)	AP011113
			Hinge connector of long tail fiber proximal connector pSs1_00253 [*Shigella* phage pSs-1]	361/371 (97%)	KM501444
HY01_0239	Putative hinge connector of long tail fiber distal connector	153598..154254	Hinge connector long tail fiber pSs1_00254 [*Shigella* phage pSs-1]	217/218 (99%)	KM501444
			Hinge connector of long tail fiber distal connector F412_gp028 [*E*. *coli* O157:H7 phage wV7]	216/218 (99%)	HM997020
HY01_0240	Possible long tail fiber distal subunit	154263..157001	Long tail fiber distal subunit RB69p254 [*E*. *coli* phage RB69]	650/679 (96%)	AY303349
			Long tail fiber distal subunit RB27_244 [*E*. *coli* phage RB27]	626/679 (92%)	KM607000
HY01_0241	Putative receptor recognition protein	157033.157812	Receptor recognition protein pSs1_00256 [*Shigella* phage pSs-1]	256/259 (99%)	KM501444
			Receptor recognition protein AR1_259 [*E*. *coli* O157:H7 phage AR1]	252/259 (97%)	AP011113
Locus tag	Predicted function	Location (nt)	BLASTP best matches	Identity^a^	Accession no.
HY01_0237	Putative long tail fiber proximal subunit	148539..152411	Long tail fiber proximal subunit pSs1_00252 [*Shigella* phage pSs-1]	1258/1290 (98%)	KM501444
			Phage long tail fiber proximal subunit F412_gp030 [*E*. *coli* O157:H7 phage wV7]	1243/1290 (96%)	HM997020
HY01_0238	Putative hinge connector of long tail fiber proximal connector	152420..153535	Hinge connector of long tail fiber, proximal connector AR1_256 [*E*. *coli* O157:H7 phage AR1]	363/371 (98%)	AP011113
			Hinge connector of long tail fiber proximal connector pSs1_00253 [*Shigella* phage pSs-1]	361/371 (97%)	KM501444
HY01_0239	Putative hinge connector of long tail fiber distal connector	153598..154254	Hinge connector long tail fiber pSs1_00254 [*Shigella* phage pSs-1]	217/218 (99%)	KM501444
			Hinge connector of long tail fiber distal connector F412_gp028 [*E*. *coli* O157:H7 phage wV7]	216/218 (99%)	HM997020
HY01_0240	Possible long tail fiber distal subunit	154263..157001	Long tail fiber distal subunit RB69p254 [*E*. *coli* phage RB69]	650/679 (96%)	AY303349
			Long tail fiber distal subunit RB27_244 [*E*. *coli* phage RB27]	626/679 (92%)	KM607000
HY01_0241	Putative receptor recognition protein	157033.157812	Receptor recognition protein pSs1_00256 [*Shigella* phage pSs-1]	256/259 (99%)	KM501444
			Receptor recognition protein AR1_259 [*E*. *coli* O157:H7 phage AR1]	252/259 (97%)	AP011113

^a^ amino acid sequence identity

After a phage infects the specific host strain, it generally shows bacteriocidal activity to lyse the host strain within a few hours. However, after lysis, surviving host cells can continue to grow due to the selection of bacteriophage-insensitive mutants (BIMs) [9]. The development of BIMs is problematic and should be reduced for further phage applications to control various pathogens. The temporary modification of host receptors has been suggested to be involved in phage resistance [26, 27]. *Salmonella* Typhimurium has the ability to modify the host receptor for transient resistance against phage SPC35 infection [26]. This host strain temporally glycosylates the LPS layer to increase resistance to phage infection, and its sensitivity for phage infection recovers after sub-culturing, substantiating transient phage resistance via temporary modification of host receptors. To reduce the development of BIMs, three methods are suggested: (1) identifying phages containing multiple genes associated with the recognition of various host receptors, (2) genetic manipulation of phages to carry these host receptor-associated genes in the phage genome, and (3) development of phage cocktails containing various phages recognizing different host receptors. Previously, the phage cocktail approach was used to reduce the emergence of BIMs with three different *Pseudomonas aeruginosa* phages (Pa1, Pa2, and Pa11) recognizing different *P*. *aeruginosa* host receptors in the mouse model, showing significant protection with over 87% survival of *P*. *aeruginosa-*infected mice [28].

Comparative genome analyses of phages have increased the understanding of their genomic characteristics as well as evolutionary relationship among related phages. Comparative dot plot analysis with whole phage genomes (Fig 6A) and phylogenetic tree analysis with genes encoding the phage terminase large subunit (Fig 6B) revealed the presence of Group I including *E*. *coli* O157:H7, *S*. *flexneri*, and *Yersinia* phages, suggesting that these phages are closely related and evolved from a common ancestor. Subsequent comparative whole genome analyses of closely-related phages, such as HY01, *E*. *coli* phage T4, *E*. *coli* O157:H7 phage AR1, *Shigella* phage pSs-1, and *S*. *flexneri* phage Shfl2 in Group I, were conducted. Genome comparison of HY01, T4, and AR1 showed that two genes encoding the long tail fiber distal subunit and receptor recognition protein are shared with *E*. *coli* O157:H7-infecting phages HY01 and AR1 with high sequence identity, but *E*. *coli*-infecting phage T4 has only one fused gene and little or no sequence identity with these two genes of HY01, suggesting that they recognize and infect different host strains (Fig 7A and 7B). Actually, the hosts of HY01 and AR1 include *E*. *coli* O157:H7 strains, whereas the known host strain of T4 is non-O157:H7 *E*. *coli* (Table 1); therefore, these two genes are probably critical for the determination of host range.

Many phage products have been announced since the US FDA approval of phages for applications as natural food preservatives. Because phage HY01 has the advantage of controlling *E*. *coli* O157:H7 and *S*. *flexneri* simultaneously, its biocontrol ability should be verified in food samples for the development of a novel natural food preservative. In addition, based on its complete genome sequence analysis, it lacks genes encoding toxins and virulence factors, suggesting its safety for human consumption (Fig 5). The application of phage HY01 to fresh edible cabbage samples contaminated with clinical and food isolates of *E*. *coli* O157:H7 showed that these pathogens were possibly controlled up to 2 h (food isolate) or 4 h (clinical isolate) (Fig 10). However, the *E*. *coli* O157:H7 host strain demonstrated regrowth, probably due to incomplete lysis or development of BIMs. Therefore, the regrowth problem of the host strain after phage treatment should be answered for further food applications of phage HY01. In addition, the low MOI of HY01 did not inhibit food or clinical isolates under food conditions, while the challenge assay with an MOI of 10 functioned well for biocontrol of *E*. *coli* O157:H7 under laboratory conditions. This may be because the large amount of food matrices reduces or hinders the opportunity for HY01 to encounter the test strain. To avoid food matrix hindrance, the MOI value was increased to 10^6^, which showed HY01 inhibition activity. Because of this high MOI, food applications with HY01 may not be practical, and the high MOI is another hurdle to overcome for real food applications. Nevertheless, reduction of viable host cell counts by the phage HY01 indicates bacteriocidal activity, suggesting that this phage may be a candidate for the development of a novel food preservative against *E*. *coli* O157:H7 in food environments. However, the growth recovery of both food and clinical isolates in foods after phage inhibition was observed. To overcome this problem, an alternative phage cocktail approach with various *E*. *coli* O157:H7 phages using different host receptors for infection may be recommended for further food applications.
